# Ultrastructural Changes Associated With the Enhanced Permeability of the Round Window Membrane Mediated by Ultrasound Microbubbles

**DOI:** 10.3389/fphar.2019.01580

**Published:** 2020-01-28

**Authors:** Yi-Chun Lin, Hsin-Chien Chen, Hang-Kang Chen, Yuan-Yung Lin, Chao-Yin Kuo, Hao Wang, Chia-Lien Hung, Cheng-Ping Shih, Chih-Hung Wang

**Affiliations:** ^1^ Graduate Institute of Medical Sciences, National Defense Medical Center, Taipei, Taiwan; ^2^ Department of Otolaryngology-Head and Neck Surgery, Tri-Service General Hospital, National Defense Medical Center, Taipei, Taiwan; ^3^ Teaching and Research Section, Taichung Armed Forces General Hospital, Taichung, Taiwan; ^4^ Taichung Armed Forces General Hospital, Taichung, Taiwan

**Keywords:** ultrasound, microbubble, round window membrane (RWM), permeability, inner ear, ultrastructure, scanning electron microscope (SEM), transmission electron microscope (TEM)

## Abstract

The round window membrane (RWM) is the most common entryway for local drug and gene delivery into the inner ear, but its permeability can change the treatment outcome. We previously demonstrated a feasible and highly efficient approach using ultrasound-aided microbubble (USMB) cavitation to enhance the permeability of the RWM. Here, we investigated the safety of USMB exposure and the association between temporal changes in RWM permeability and ultrastructure. Experimental guinea pigs were divided into two treatment groups: a control group receiving round window soaking (RWS) with MBs and treatment (USM) groups undergoing 3 (USM-3) or 5 (USM-5) consecutive USMB exposures (1 min/exposure) at an acoustic intensity of 3 W/cm^2^ and 1 MHz frequency. The trans-RWM delivery efficiency of biotin-fluorescein isothiocyanate conjugates, used as permeability tracers, revealed a greater than 7-fold higher delivery efficiency for the USM groups immediately after 3 or 5 exposures than for the RWS group. After 24 h, the delivery efficiency was 2.4-fold higher for the USM-3 group but was 6.6-fold higher for the USM-5 group (and 3.7-fold higher after 48 h), when compared to the RWS group. Scanning electron microscopy images of the RWM ultrastructure revealed USMB-induced sonoporation effects that could include the formation of heterogeneous pore-like openings with perforation diameters from 100 nm to several micrometers, disruption of the continuity of the outer epithelial surface layer, and loss of microvilli. These ultrastructural features were associated with differential permeability changes that depended on the USMB exposure course. Fourteen days after treatment, the pore-like openings had significantly decreased in number and the epithelial defects were healed either by cell expansion or by repair by newly migrated epithelial cells. The auditory brainstem response recordings of the animals following the 5-exposure USMB treatment indicated no deterioration in the hearing thresholds at a 2-month follow-up and no significant hair cell damage or apoptosis, based on scanning electron microscopy, surface preparations, and TUNEL assays. USMBs therefore appear to be safe and effective for inner ear drug delivery. The mechanism of enhanced permeability may involve a disruption of the continuity of the outer RWM epithelial layer, which controls transmembrane transport of various substances.

## Introduction

**Figure 1:**
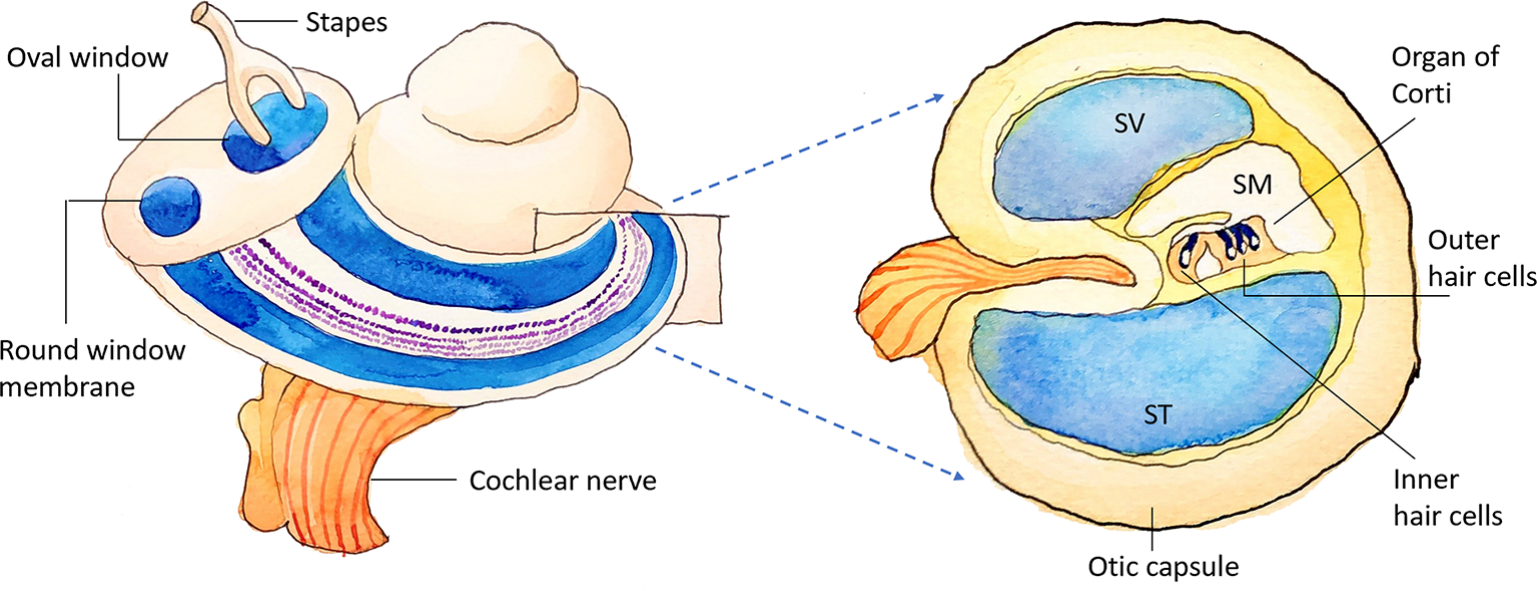
Schematic illustration of the inner ear. SV, scala vestibuli; SM, scala media; ST, scala tympani.

The delivery of drugs and genes into the human inner ear remains a current challenge, not only because of the anatomically complex structure of the inner ear but also due to its inaccessibility and vulnerability as a sensory organ (**Figure 1**). Although several inner ear diseases can be managed with the systemic drug administration, the pre-existing blood-labyrinth barrier and the potentially adverse effects of systemic medication all hamper an effective therapeutic dosage from reaching the inner ear. Local administration provides the advantage of precise targeting and avoids the risk of systemic adverse events (El Kechai et al., 2015; Li et al., 2017; Hao and Li, 2019; Piu and Bishop, 2019). The methods of local drug administration to the inner ear include intratympanic and intracochlear approaches, where the latter offers a direct delivery route to achieve a greater drug bioavailability by either penetrating right through the round window membrane (RWM) or through an opening in the cochlear bony wall (El Kechai et al., 2015; Mäder et al., 2018). However, the intracochlear approach poses a high risk of inner ear damage and hearing loss and is therefore mainly employed as a combined procedure during cochlear implant surgery (Mccall et al., 2010; Chin and Diaz, 2019).

Intratympanic medication injection, by way of the RWM as a transfer site for medication delivery into the cochlea, is currently the most common clinical procedure used for inner ear drug delivery. For example, intratympanic injections of corticosteroids are effective for treating sudden sensorineural hearing loss, and aminoglycoside injection is used to treat Meniere’s disease (Jackson and Silverstein, 2002; Rauch et al., 2011; Li et al., 2017). These injection procedures are minimally invasive; however, effective therapeutic outcomes may require repeated injections, avoidance of swallowing by the patient, or placing the patient’s head slightly lower than the body because medications in the middle ear cavity usually drain out through the Eustachian tube (Shih et al., 2019). To overcome the short residence times for contact with the RWM, several delivery devices, such as the MicroWick, microcathers, and osmotic pumps, have been developed to prolong the duration of medication contact with the RWM. Delivery materials and agents such as gelfoam, hyaluronic acid hydrogels, histamine, nanoparticles, and nanovesicles can also provide a sustained inner ear delivery *via* the RWM (El Kechai et al., 2015; Mäder et al., 2018; Creber et al., 2019).

Recently, ultrasound (US) combined with a microbubble (MB) contrast agent has been demonstrated to target or control drug release to tissues and cells (Tang et al., 2002; Smith et al., 2003; Hernot and Klibanov, 2008). In 2013, our research team demonstrated that trans-membrane drug delivery into the inner ear can be assisted and enhanced by sonophoresis with US-aided MBs (USMBs) (Shih et al., 2013). This technique not only increases the permeability of the RWM and facilitates drug delivery into the inner ear, but the preliminary results also show no resulting damage to the integrity of the RWM or deterioration of the hearing thresholds, as assessed by auditory brainstem responses. Furthermore, in 2014, we demonstrated that USMBs were effective at facilitating gene transfer to auditory cells *in vitro* (Liao et al., 2014) and that the size-dependent MB oscillation behavior in the presence of US plays a role in enhancing gene transfer. In addition, dexamethasone delivery to the round window of animals with the aid of USMBs has a greater efficacy in protecting the inner ear from noise-exposed injury when compared to a simple soaking with the drug (Shih et al., 2019).

In the cochlea, the RWM not only serves a membranous barrier between the inner ear and the middle ear cavity, but it also provides the main route for local drug and gene delivery into the inner ear. The RWM is made up of an outer epithelial layer, a middle connective tissue layer, and an inner epithelial layer (Goycoolea and Lundman, 1997). Of these three layers, the outer epithelial layer is believed to prevent the passage of substances from the middle ear to the inner ear. Substance transport across the RWM can involve several cellular processes: diffusion down a concentration gradient, pinocytosis, or transcellular movement through channels. The anatomical characteristics of the outer epithelium include absorbent microvilli and lateral interdigitations, tight junctions between cells, a continuous basement membrane, abundant mitochondria and rough endoplasmic reticulum, and a well-developed Golgi complex (Paparella et al., 1983; Goycoolea and Lundman, 1997; Goycoolea, 2001). All these features contribute to the permeability of the RWM (Goycoolea et al., 1988).

Our previous studies on animal models suggested that the USMB technique is both practical and successful. However, translating this technique to the clinic requires exploration of the USMB-induced RWM permeability changes and identification of any safety issues. The aim of the present study was therefore to evaluate a possible association between the number of USMB exposure courses, the permeability changes in the targeted RWM and its related ultrastructural alterations, and the safety concerns regarding application of USMBs to the middle ear cavity for inner ear drug delivery.

## Materials and Methods

### Animals and Study Design

**Figure 2:**
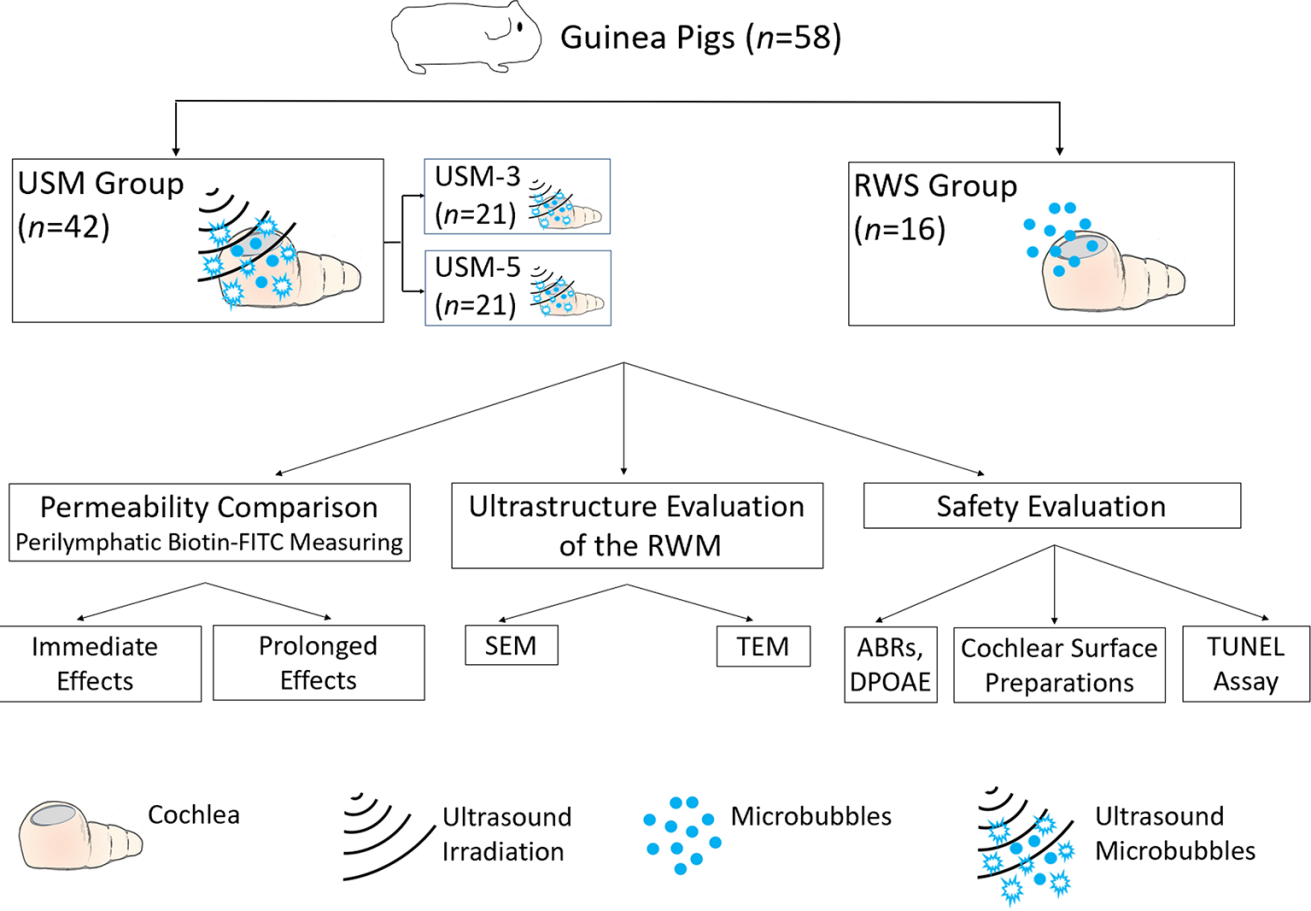
Flow chart of the study design and animal groups. USM, ultrasound microbubble treatment; RWS, round window membrane soaking treatment; RWM, round window membrane; SEM, scanning electron microscopy; TEM, transmission electron microscopy; ABR, auditory brainstem response; DPOAE, distortion product otoacoustic emission; TUNEL, terminal deoxynucleotidyl transferase dUTP nick end labeling.

Guinea pigs with normal Preyer’s reflex, weighing 250–350 g, were divided into two groups: 1) an ultrasound microbubble (USM) group: animals receiving 3 (USM-3) or 5 (USM-5) courses of USMB application and 2) a round window soaking (RWS) group that served as the control and received MBs soaked into the tympanic bulla. In the USM groups, the animal’s tympanic bulla was filled with 200 µl of MBs, followed by three or five consecutive US exposures. In the RWS group, the animal’s tympanic bulla was filled as above with the same volume of MBs but no US treatment was given. After the treatment, the cochleae of all animals were soaked for 10 min with biotin-fluorescein isothiocyanate conjugates (FITC; 40 µg/ml) at each of the examined time points. The collected samples were evaluated for permeability and ultrastructure of the RWM, and the safety of the procedure was tested by auditory brainstem response (ABR) and distortion product otoacoustic emission (DPOAE) evaluations. Under this protocol, a total of 58 animals were tested, including technical failures. A flow chart of the study design is presented in **Figure 2**.

### Microbubble Preparation and Ultrasound Exposure

SonoVue^®^ (Bracco, Milano, Italy) phospholipid MBs containing sulfur hexafluoride were freshly reconstituted prior to use by mixing the lyophilizate with 5 ml of 0.9% saline to form a suspension containing 2−5 × 10^8^ bubbles/ml. The ultrasound device (ST2000V, Nepa Gene, Chiba, Japan) equipped with a 6-mm-diameter transducer was used for irradiation. The optimal US exposure settings had been predetermined in our previous report (Shih et al., 2019). Briefly, the mode was set as follows: frequency 1 MHz, burst rate 250 Hz, burst duration 2 ms, acoustic intensity 3 W/cm^2^ (mechanical index [MI] = 0.254) for three or five consecutive 1-min courses; and a 50% duty cycle. The transducer was positioned at the level of the mastoid bone with opened tympanic bulla, which was at least 5 mm away from, but in alignment with, the RWM.

### Surgery

**Figure 3:**
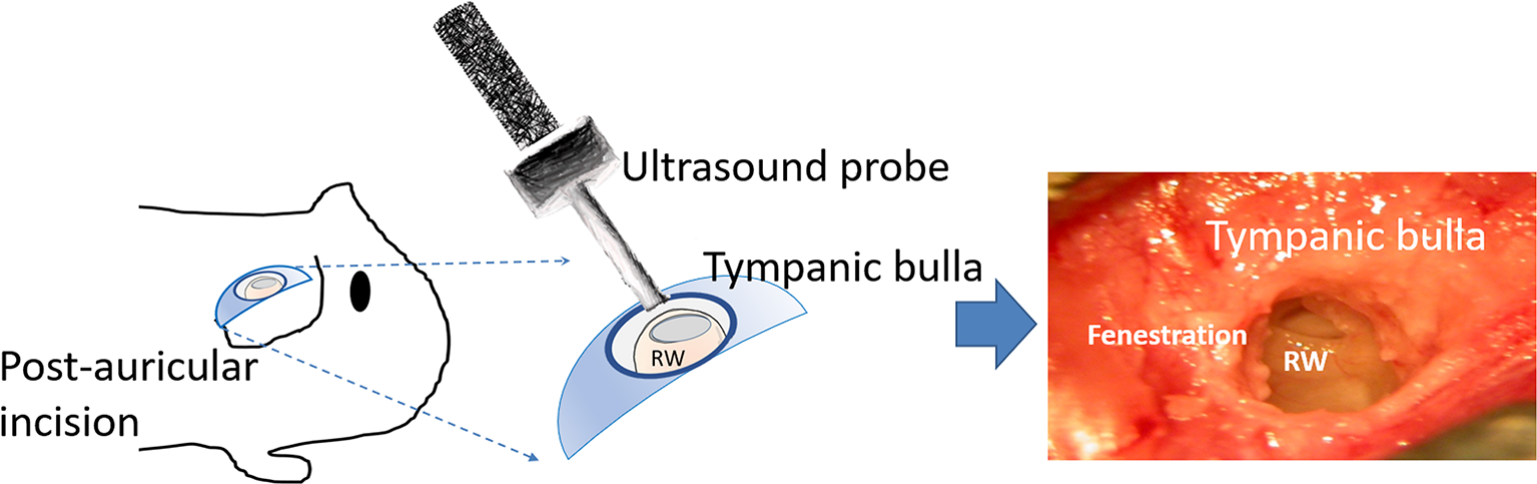
Schematic illustration of the surgery and ultrasound exposure. RW, round window.

As described and schematically illustrated in our previous study (Shih et al., 2019), guinea pigs were administered xylazine i.m. (Rompun; Bayer, Leverkusen, Germany) at 10 mg/kg and ketamine (Imalgene; Merial, Lyon, France) at 80 mg/kg. After making a post-auricular skin incision, a 4-mm-diameter fenestration was created in the tympanic bulla by drilling to expose the cochlea and round window under an operating microscope (F-170; Carl Zeiss, Germany). Ultrasound irradiation was then applied to the MBs filling the bulla through the bony fenestration (**Figure 3**). At the end of the final US exposure, the MBs were removed from both the USM and RWS groups. For immediate permeability comparisons, 200 µl of biotin-FITC was filled into tympanic bulla and soaked for 10 min. For permeability comparisons at other time points, the USM animal’s surgical wound was sutured by layers. At each examined time point, the closed surgical wound was re-opened, the tympanic bulla was filled with same volume of biotin-FITC, and soaked for 10 min.

### Perilymphatic Fluid Collection and Biotin-FITC Fluorescence Measurements

To assess the amount of biotin-FITC delivered to the cochlea, the guinea pigs were euthanized using CO_2_ gas and their cochleae were harvested from within the tympanic bulla. A 10-µl pipette microtip was gently inserted through a cochleostomy inferior to the RWM for perilymphatic fluid collection (Shih et al., 2013). The aspirated perilymphatic samples were centrifuged immediately and stored at −80°C until used for fluorescence intensity analysis using a fluorometer (excitation/emission: 485/528 nm; Fluoroskan Ascent FL, Thermo Labsystems, Finland).

### Cochlear Surface Preparation

After deep anesthesia with intraperitoneal injection of pentobarbital sodium 100 mg/kg, the animals were transcardially perfused with 4% paraformaldehyde in phosphate buffered saline (PBS; ChemCruz, Santa Cruz Biotechnology, Inc., Dallas, TX) and the cochleae were removed from the tympanic bulla and post-fixed with the same fixative at 4°C overnight, followed by dissection under a dissecting microscope to excise the cochlear lateral wall and Reissner’s membrane. The Corti sensory epithelium was permeabilized with 0.3% Triton X-100 and stained with 2% Alexa Fluor 488-conjugated phalloidin (Molecular Probes/ThermoFisher Scientific, Waltham, MA) to confirm hair cells, while 4,6-diamidino-2-phenylindole (DAPI; Molecular Probes/ThermoFisher Scientific; 5 mg/ml) was used to stain the nuclei. The entire length of the Corti sensory epithelium was cut into pieces and examined with a confocal laser scanning microscope (LSM880, Zeiss, Oberkochen, Germany).

### Scanning Electron Microscopy (SEM)

The removed cochleae were placed in fixative (2.5% glutaraldehyde with 2% paraformaldehyde in 0.1% sodium cacodylate buffer) overnight at 4°C, and then given three 10 min washes with cold PBS (0.1 M, pH 7.4). For RWM preparation, the specimens were trimmed, leaving the RWM tissue intact. Samples were then given three 15 min washes with 0.1 M cacodylate buffer containing 7% sucrose. After post-fixing in 1% osmium tetroxide (Electron Microscopy Science) and 1% thiocarbohydrizide (TCH; EMS) for 2 h, the samples were again given three 15 min washes with 0.1 M cacodylate buffer containing 7% sucrose. The specimens were dehydrated through a graded ethanol series (35%–to absolute ethanol) at 10 min intervals and then finished in a critical point dryer. The processed specimens were viewed and photographed using a SU3500 scanning electron microscope (Hitachi, Tokyo, Japan) at 15 kV.

### Transmission Electron Microscopy (TEM)

As was done for SEM, the cochleae, following fixation at 4℃ overnight and washing with cold PBS, were trimmed to leave the intact RWM soft tissue only. The whole RWM was post-fixed in 1% osmium tetroxide for 2 h, given three 15 min washes with 0.1M PBS, dehydrated in an ethanol series, and infiltrated with Spurr’s resin. The polymerized samples were sectioned with an ultramicrotome (Leica EM UC7) at a 90 nm thickness. Images were obtained using a transmission electron microscope (FEI Tecnai 20 G2 S-Twin).

### Terminal Deoxynucleotidyl Transferase dUTP Nick End Labeling (TUNEL) Assays

Paraffin-embedded cochlear sections were dewaxed in xylene and rehydrated through a graded ethanol series and double-distilled water, followed by PBS washes. Positive control sections were incubated with 100 U/ml DNase I diluted in a buffer containing 20 mM Tris-HCl (pH 7.0), 10 mM MnCl_2,_ and 1 M NaCl at room temperature for 10 min. The TUNEL assay utilizing the In Situ Cell Death Detection Kit, POD (Roche, Basel, Switzerland) was carried out following the instructions supplied by the manufacturer. Deparaffinized slides were incubated with 3% hydrogen peroxidase in methanol for 10 min to block endogenous peroxidase activity and then washed with PBS. As described in our previous report (Chen et al., 2018), the tissue sections were permeabilized first, blocked for 30 min at room temperature with the supplied blocking buffer, and then incubated with the TUNEL reaction mixture for 60 min at 37°C in a humidified atmosphere in the dark. After PBS Tween-20 (PBST) washing, the tissues were stained with Converter-POD for an additional 30 min and washed with PBST. The diaminobenzidine chromogen was then applied for 10 min to label apoptotic cells. For histological analysis, the tissue sections were counterstained with hematoxylin and viewed with an Olympus BX50 brightfield/fluorescence microscope (Olympus Corp., Tokyo, Japan) equipped with a digital camera (Olympus DP74).

### Auditory Brainstem Response (ABR) Recording

The animal’s auditory function was evaluated by recording the ABRs, as described previously (Lin et al., 2011). Briefly, guinea pigs were anesthetized and kept warm with a heating pad in a sound-attenuating chamber. Subcutaneous needle electrodes were inserted at the vertex (positive electrode), below the pinna of the ear (negative electrode), and at the back (ground electrode). Specific stimuli (clicks and 8, 16, and 32 kHz tone bursts) were generated using SigGen software (Tucker-Davis Technologies, Alachua, FL) and delivered *via* an earphone inserted into the external auditory canal. The average responses from 1,024 stimuli for each frequency were obtained by reducing the sound intensity in 5-dB steps until reaching a threshold. The resulting ABR thresholds were defined as the lowest intensity at which a reproducible deflection in the evoked response trace could be recognized.

### Distortion Product Otoacoustic Emission Measurements

The distortion product otoacoustic emissions (DPOAEs) were measured at the center frequencies (FCs) of 8, 16, 20, 24, and 32 kHz with a Real-time Signal Processing System (Tucker-Davis Technologies), as reported previously (Chen et al., 2018). Briefly, two simultaneous continuous pure tones, F1 and F2 were calculated using the FC to yield a frequency of primary 1 (Tone 1) and primary 2 (Tone 2). The two primary tones were presented at the same intensity (L1 = L2 = 65 dB SPL) and at a frequency ratio (F2/F1) of 1.2. The primary tones produced by two separate speakers (EC1 close-field speakers; Tucker-Davis Technologies) were introduced into the animal’s ear canal. The DPOAE recordings were made with a low-noise microphone (ER 10B; Etymotic Research, Elk Grove Village, IL) and averaged 512 times at each frequency. The peak of the cubic difference distortion product (2F1 − F2) at different FCs was accepted as a DPOAE if it was 3 dB above the noise floor, and the difference was referred to as the signal-to-noise ratio (SNR).

### Monitoring of Cochlear and Tympanic Cavity Temperature Changes

The temperature changes in the cochlea and tympanic cavity after various courses of USMB exposures were monitored using a thermometer coupled to a fine sensor probe (Center-301 type K; CENTER Technology Corp., New Taipei City, Taiwan), with a resolution of 0.1°C. The sensor probe was inserted at different depths into the tympanic cavity filled with MBs and would concomitantly touch the nearby cochlea to measure the temperature before USMB treatment and at the end of various USMB courses. The three designed locations for temperature measurements, from the top to the bottom of the tympanic cavity, were the cochlear basal turn near the RWM, the middle turn, and the apex. Temperature measurements began at the cochlea, then in the tympanic cavity, and then at the cochlea and were processed alternately. The temperature differences between the two measurements were recorded.

### Statistical Analysis

Statistical analysis was performed using a two-tailed Student’s *t* test for comparison of the means between two groups and the Kruskal-Wallis test or one-way ANOVA with *post hoc* Bonferroni correction for multigroup comparisons. Data were expressed as mean ± standard error of the mean.

## Results

### Permeability Changes in the RWM Depend on the Number of Exposures to USMBs

**Figure 4:**
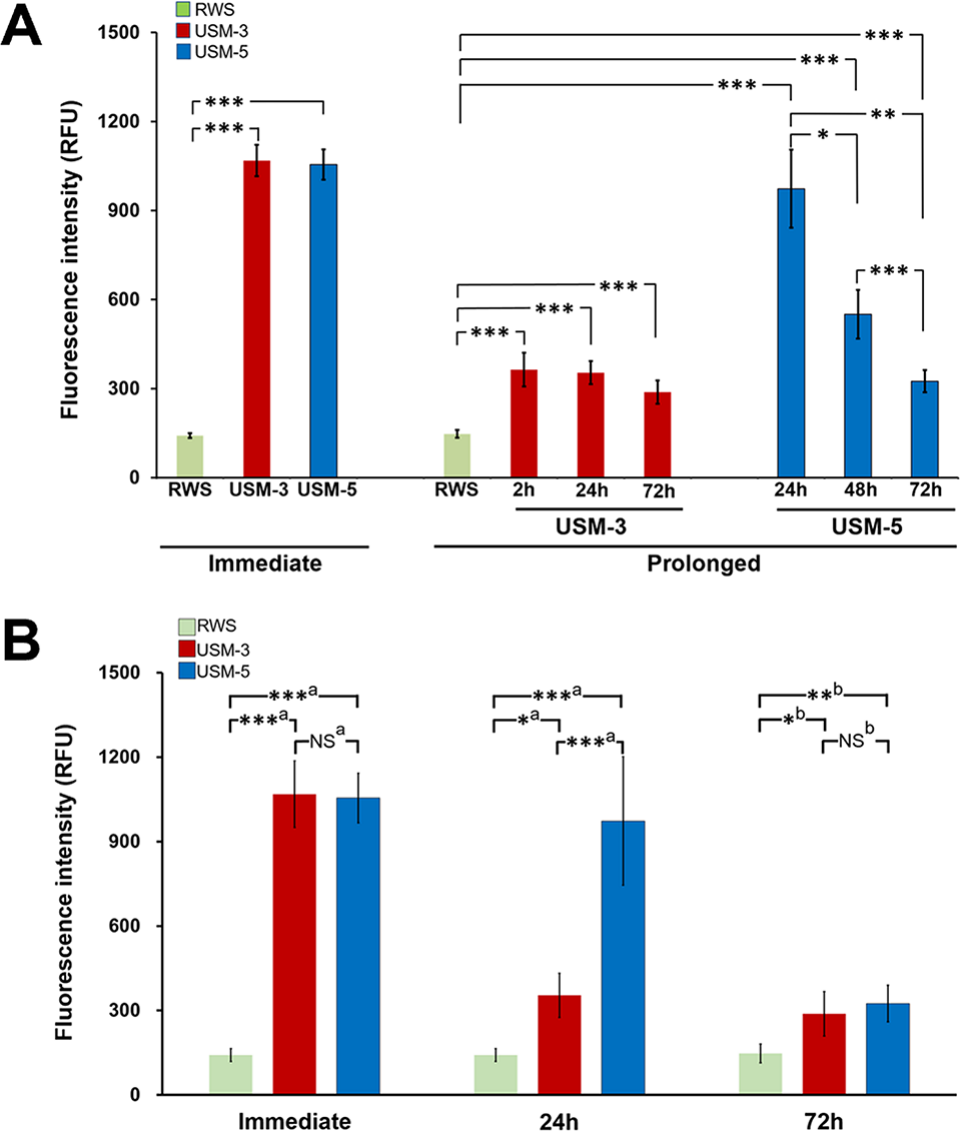
Evaluation of the permeability changes of the round window membrane (RWM) following three or five ultrasound microbubble (USMB) treatments and a 10-min biotin-fluorescein isothiocyanate solution soaking of the tympanic bulla immediately after USMB treatment and at different time points post USMB treatment. **(A)** The bars indicate the mean maximum fluorescence in relative fluorescence units (RFU) of the assessed USM-3 (red) and USM-5 (blue) groups when compared with the control RWS group (green). **(B)** Multigroup comparisons using the Kruskal-Wallis test or one-way ANOVA, followed by *post hoc* Bonferroni tests. Values are expressed as mean ± SEM; n = 4 per group. a = Kruskal-Wallis test with Bonferroni correction; b = one-way ANOVA test with Bonferroni correction; USM, ultrasound microbubble treatment; RWS, round window soaking with microbubbles treatment; *p < 0.05, **p < 0.01, ***p < 0.001, NS, not significant.

We first examined the USMB-mediated RWM permeability changes by comparing the perilymphatic levels of delivered biotin-FITC at different time points. As shown in **Figure 4**, comparison of the fluorescence intensity of the USM-3 and control groups at each time point, immediate (1068.4 ± 53.0 vs. 141.6 ± 8.5, *p* < 0.001), 2 h (363.9 ± 56.9 vs. 147.3 ± 13.5, *p* < 0.01), 24 h (353.7 ± 39.1 vs. 147.3 ± 13.5, *p* < 0.01), and 72 h (288.1 ± 39.3 vs. 147.3 ± 13.5, *p* < 0.01) revealed a significantly enhanced permeability of the RWM in the USM-3 group compared to the control group. In the USM-3 group, the measured fluorescence intensity showed its highest level (7.5-fold higher than control) at the 10 min time point immediately post USMB exposure, followed by a gradual decrease in fluorescence levels at subsequent post exposure time points (2.5-fold at 2 h, 2.4-fold at 24 h, and 2.0-fold at 72 h vs. the control) (**Table 1**).

**Table 1:** Comparison of the efficiency of USMB exposure courses for inner ear drug delivery.

Time points post USMB	Exposure number of USMB	Delivered Biotin-FITC Fluorescence Intensity	Fold-increase of inner ear delivery (relative to control)
*Immediate*			
	3	1068.4 ± 53.0	7.5
	5	1055.0 ± 51.0	7.4
	Control	141.6 ± 8.5	–
*Prolong*			
2 h	3	363.9 ± 56.8	2.5
24 h	3	353.7 ± 39.1	2.4
72 h	3	288.1 ± 39.3	2.0
24 h	5	973.3 ± 131.6	6.6
48 h	5	550.5 ± 82.3	3.7
72 h	5	324.7 ± 37.3	2.2
72 h	Control	147.3 ± 13.5	–

In the USM-5 group, the delivered biotin-FITC level was also significantly higher at each time point immediately after USMB treatment and after 72 h when compare to the control (**Figure 4**). Like the USM-3 group, the USM-5 group also demonstrated a post-USMB time-dependent permeability change of the RWM as shown by the gradually decreasing delivery of biotin-FITC from immediately after the USMB treatment to 72 h later. These data suggest that USMBs can effectively enhance the permeability of the RWM and that the enhancement could be maintained for at least 72 h. Five consecutive treatments with USMBs caused a similar immediate transmembrane delivery effect to that observed with 3 treatments (1055.0 ± 51.0 vs. 1068.4 ± 53.0, *p =* 0.87); however, after 24 h, the 5-course USMB treatment delivered a higher level of biotin-FITC than the 3-course treatment (973.3 ± 131.6 vs. 353.7 ± 39.1, *p* = 0.004), suggesting that 5 courses of USMB treatment may sustain a more enhanced permeability change in the RWM.

### Ultrasound-Mediated MB Cavitation and Sonoporation on the Outer Epithelial Layer of the RWM Enhanced Permeability

**Figure 5:**
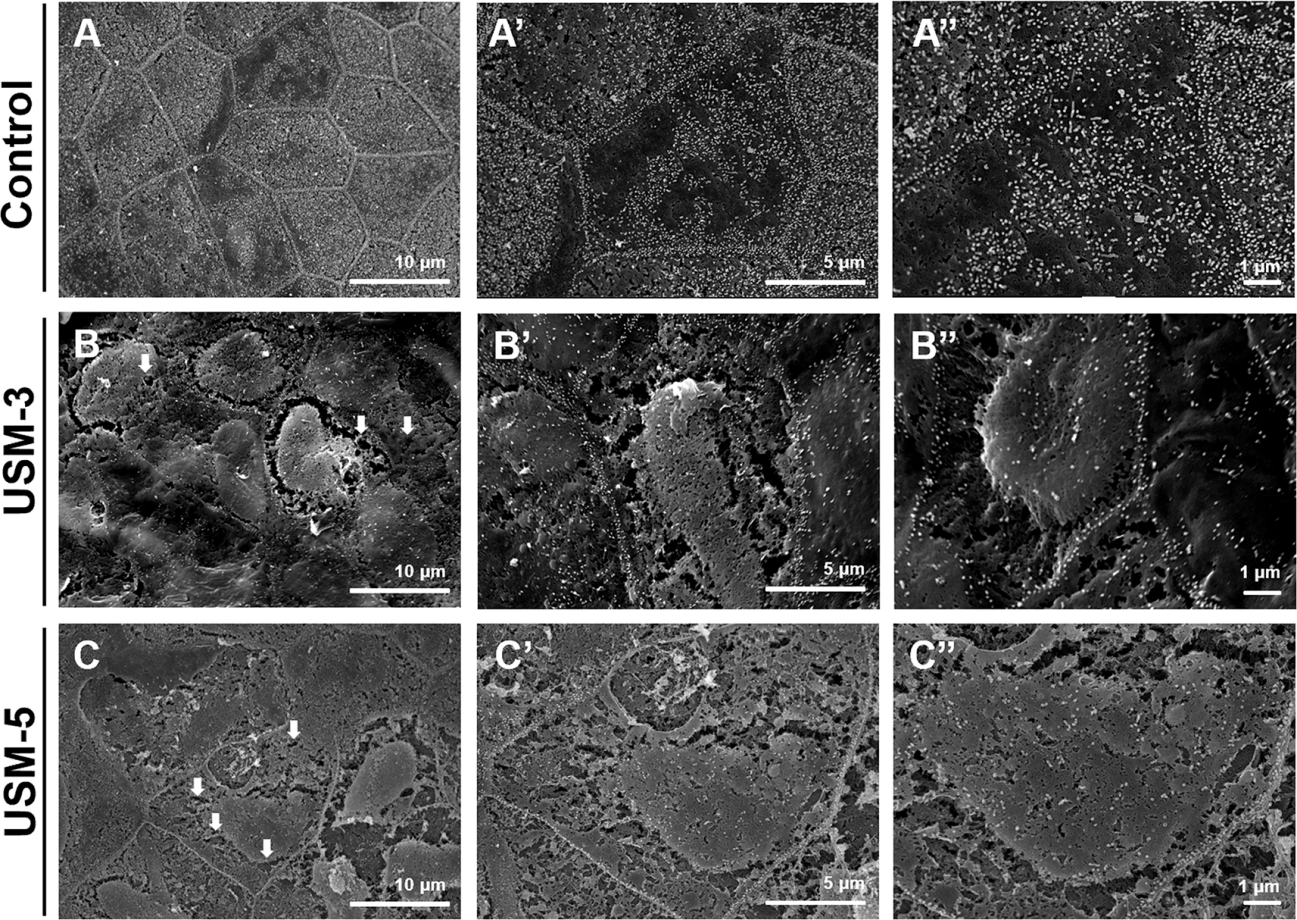
Scanning electron microscopy (SEM) view of the epithelial surface of the round window membrane (RWM) under different magnifications. **(A−A”)** RWM from a control animal after microbubbles (MBs) soaking for 10 min without ultrasound (US) exposure. **(B−B”)** Samples were immediately taken from the animal after three ultrasound microbubble (USMB) treatments. **(C−C”)** After five USMB treatments. The white arrows indicate pore-like defects.

SEM examination of the sequential changes of the RWM ultrastructural features at different time points after USMB treatments revealed a normal architecture of the outer epithelium in the control animals, with flat cells arranged in pentagonal or octagonal patterns and abundant microvilli (**Figures 5A−A”**). The RWM in the USM-3 group immediately after USMB treatment showed various degrees of heterogeneous pore-like openings, with sizes from 100 nm to several microns, on the epithelial surface. Some areas even showed separation of the epithelial cells, with fissures appearing on the cell boundaries where the tight junctions between adjacent cells were originally located (**Figures 5B−B”**). The USM-5 samples showed more extensive pore-like defects and disruption of the continuity of the cell membrane on the epithelial surface, as well as a significant loss of microvilli (**Figures 5C−C”**). All these observations suggest a direct involvement of cavitation-enhanced sonoporation on the targeted RWM.

**Figure 6:**
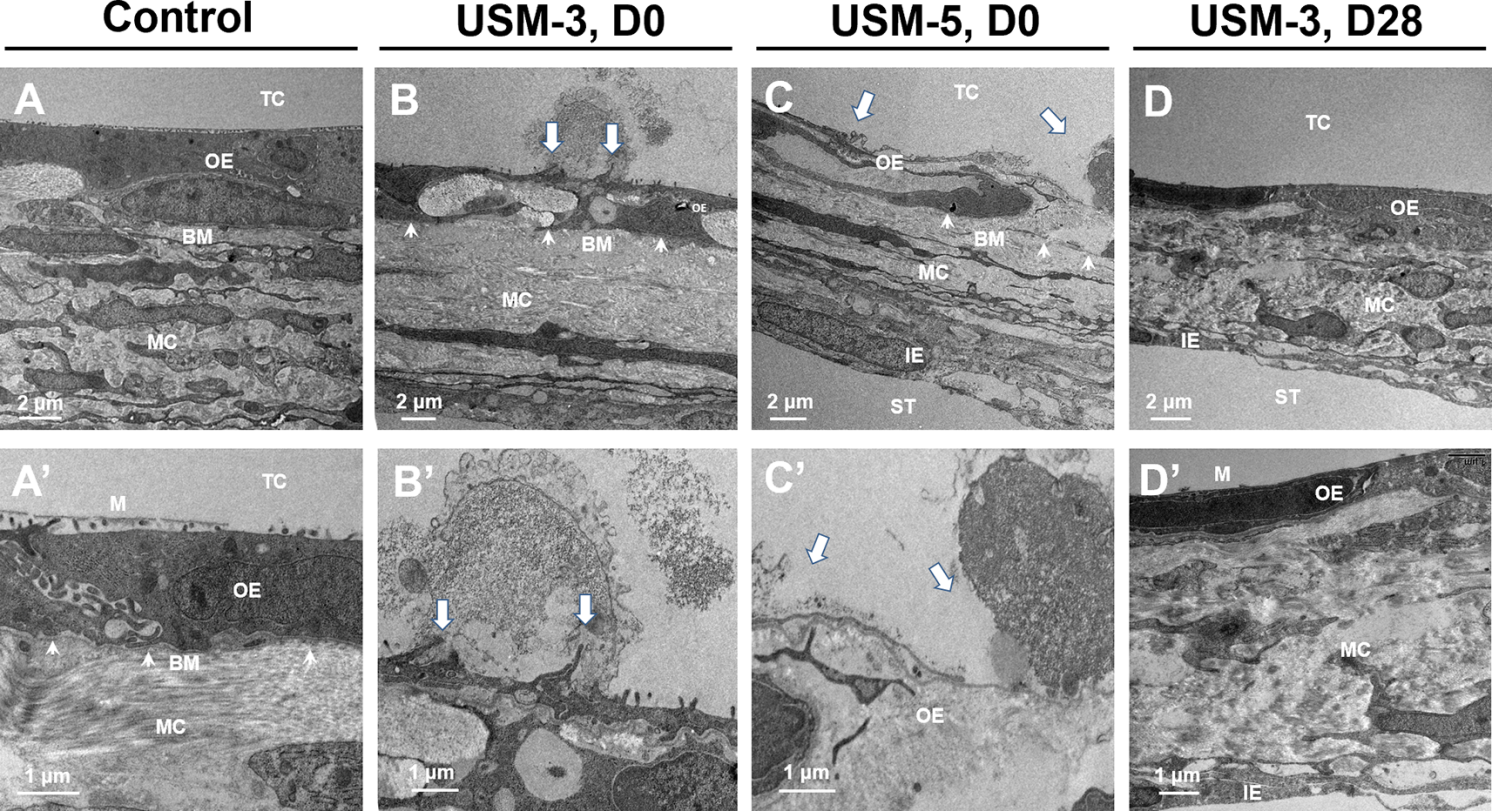
Cross-sectional transmission electron microscope (TEM) images of the round window membrane (RWM) under different magnifications. **(A−A’)** RWM from the control animal after soaking with microbubbles (MBs) for 10 min without ultrasound (US) exposure. **(B−B’)** Samples immediately taken from animal after three ultrasound microbubble (USMB) treatments. **(C−C’)** Samples after five USMB treatments. **(D−D’)** Samples taken from animals 28 days after receiving 3 courses of USMB treatment. The white arrows indicate outer epithelial membrane defects. The white arrowheads indicate basement membrane. TC, tympanic cavity; OE, outer epithelial layer; MC, middle connective tissue layer; IE, inner epithelial layer; BM, basement membrane; ST, scala tympani; M, microvilli; D0, day 0; D28, day 28.

**Figure 6** shows the TEM views of cross-sections of the RWM after USMB. Microbubble cavitation resulted in various degree of disruption on the outer epithelial cells, including the formation of pits of different sizes and rising of the cell membranes (**Figures 6B−B’**, arrows). Extensive disruptions of the outer epithelial cells and membrane defects were noted in the USM-5 group (**Figures 6C−C’**, arrows). However, the sonoporation effects seemed to disturb only the outer epithelial layer of the RWM, because the basement membrane along the outer epithelial cells remained intact (**Figures 6B−C**, arrowheads).

### Post-Cavitation Epithelial Wound Healing

**Figure 7:**
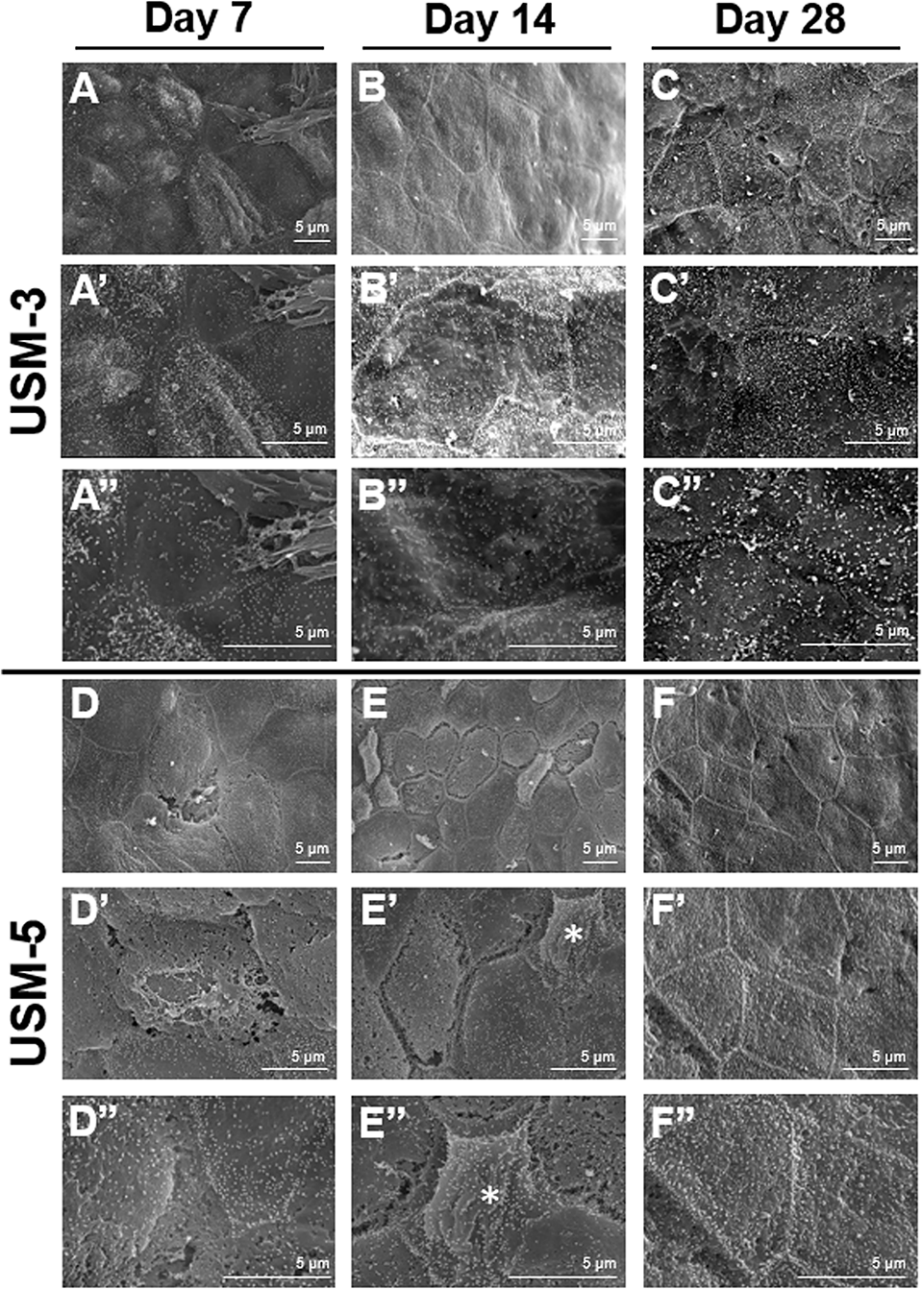
Scanning electron microscopy (SEM) view of the epithelial surface of the round window membrane (RWM) under different magnifications at different time points post ultrasound microbubble (USMB) treatment. **(A−A”)** RWM after 3 courses of USMBs for 7 days, **(B−B”)** 14 days, and **(C−C”)** 28 days. **(D−D”)** Samples after 5 courses of USMBs for 7 days, **(E−E”)** 14 days, and **(F−F”)** 28 days.

A series of SEM images taken at different time points after USMB treatment demonstrated outer epithelial barrier disruption (**Figure 7**). On day 7 after USMB treatment, the previously sonoporation-induced breaches between adjacent cells began to fill up in the USM-3 group (**Figures 7A−A”**), whereas many gaps remained in the USM-5 group (although the size and area of the gaps had significantly reduced) (**Figures 7D−D”**). On day 14 after USMB treatment, the epithelial wounds in the USM-3 group had almost fully healed with a cell-expansion-like pattern (**Figures 7B−B”**), whereas regenerative epithelial cell migration was observed in the USM-5 group and had begun to cover the wound area (**Figures 7E−E”** **, asterisk)**. By day 28, microvillus regrowth was evident on the outer epithelial surface of the USM-3 group (**Figures 7C−C”**), the TEM images also revealed a completely recovered outer epithelial layer in the USM-3 group (**Figures 6D−D’**). By contrast, only a limited number of microvilli were found in the USM-5 group (**Figures 7F−F”**). Taken together, the results indicate that 3 or 5 courses of USMB treatment caused a reversible epithelial wounding that healed without damaging the basement membrane. These ultrastructural changes of the outer epithelium were associated with a differential permeability of the RWM that depended on the USMB exposure.

### Thermal Effects of USMBs Applied to the Tympanic Cavity

**Figure 8:**
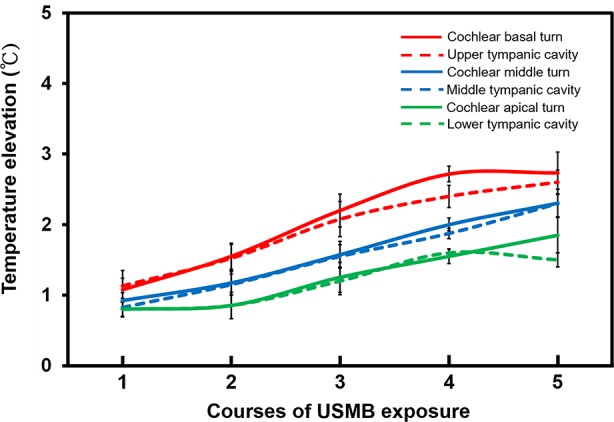
The elevation of the cochlea and tympanic cavity temperature after different courses of ultrasound microbubble (USMB) exposure. The results are expressed as the mean ± SEM; n = 5 for each point.

**Figure 8** shows the range of temperature increases for both the cochlea and the tympanic cavity after various courses of USMBs. One or two courses of USMBs resulted in a slight drift in the temperature rise of around 0.8°C–1.5°C on the cochlea and in the tympanic cavity. By contrast, three or more courses of irradiation caused rapid increases in temperature and greater heating over the cochlear basal turn and its adjacent upper tympanic cavity (2.0°C–2.7°C) than over the cochlear apical turn and the adjacent lower tympanic cavity (1.2°C–1.8°C). This finding indicated an attenuated temperature gradient in the USMB-exposed tympanic cavity, which displayed the greatest temperature elevation at the top and the least elevation at the bottom of the cavity. The temperature also tended to be lower, by about 0.2°C, in the tympanic cavity than on the cochlea after more than 3 courses of irradiation, implying a much greater heat deposition at the bone surface than in the MB solution in the cavity. These data suggest that ultrasound absorption by the bony cochlea or by the surrounding MBs solution is greatest on the exposed surface of the tissue close to the transducer face and decreases with increased propagation distance or depth.

### Preservation of Hearing Thresholds and Cochlear Integrity After USMB Treatment

**Figure 9:**
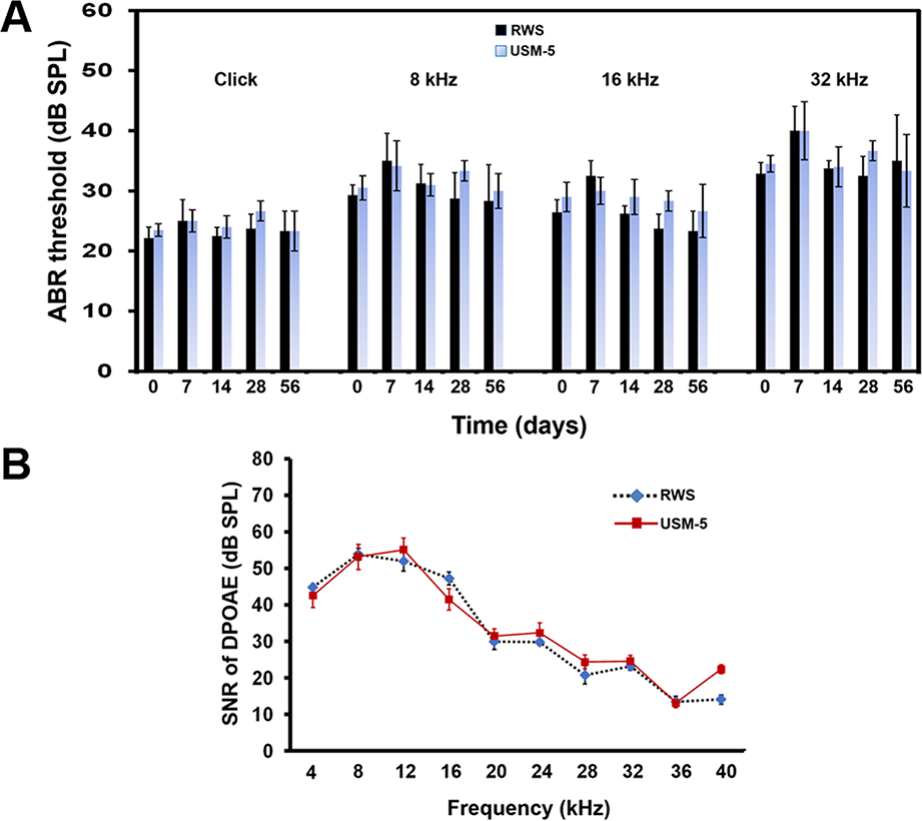
Hearing assessment in guinea pigs after 5 courses (USM-5) of ultrasound microbubble (USMB) treatments. **(A)** The auditory brainstem response (ABR) threshold recordings in the round window soaking (RWS) and USM-5 groups before (day 0) and at a two-month follow up after USMB treatments. **(B)** Signal-to-noise ratios (SNRs) of the cubic difference distortion product (2F_1_–F_2_) at different center frequencies (F_C_) for each group. The results are expressed as the mean ± standard error of the mean (SEM), with n = 4 for each bar.

We also performed ABRs on the treated animals to evaluate whether USMB intervention compromised the animals’ hearing thresholds. In an earlier paper, we described that a two-course USMB treatment did not cause hearing threshold shifts or damage to the cochlear hair cells (Shih et al., 2013). In this study, hearing assessments were only performed on animals of the RWS and USM-5 groups to reduce the number of animals used. The results of the ABR hearing assessments, to both click-evoked and tone burst-evoked sounds at a frequency of 8, 16, and 32 kHz, showed that the hearing in animals that received 5 courses of USMB treatment did not differ from that of the controls that had MBs soaking during a two-month follow up (**Figure 9A**). The signal-to-noise ratio (SNR) of the distortion product (DP) measurements immediately after 5 courses of USMB at frequencies from 4 kHz to 32 kHz among the treatment and control groups also did not differ (**Figure 9B**).

**Figure 10:**
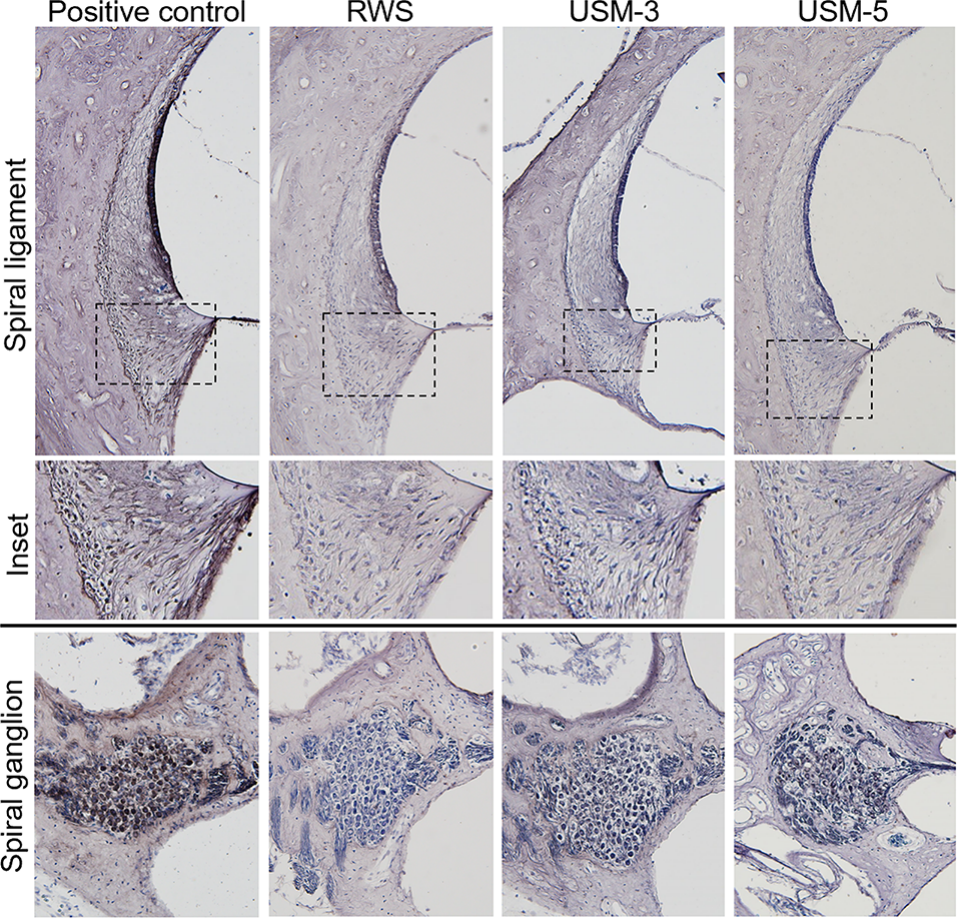
Effects of various ultrasound microbubble (USMB) courses on cell death in the target inner ear. Representative photos of the spiral ligament and spiral ganglion examined by Transferase dUTP Nick End Labeling (TUNEL) assays following round window soaking (RWS) and USMB treatment (original magnification ×200). A section treated with DNase I served as a positive control.

**Figure 11:**
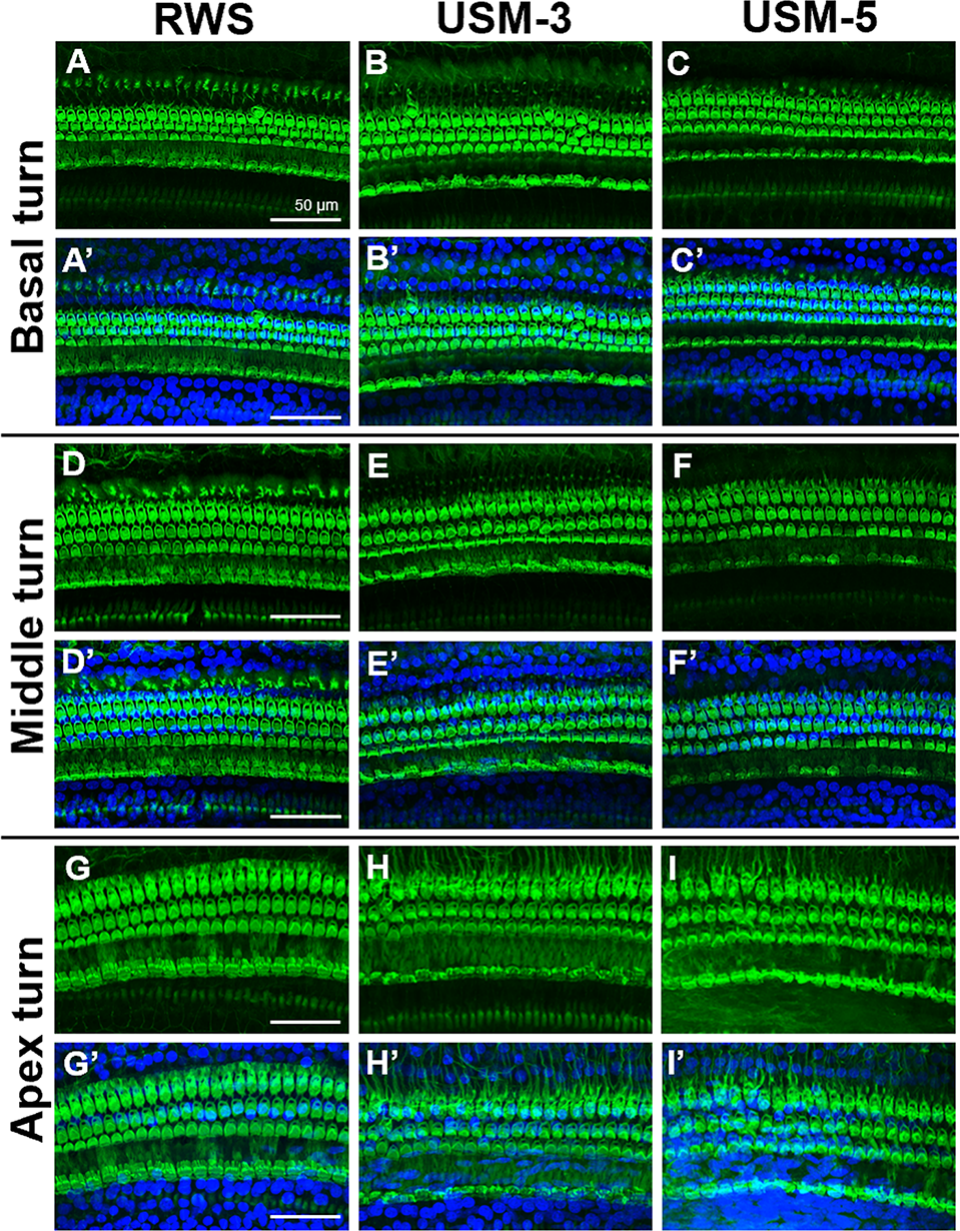
Cochlear sensory epithelial surface preparations were obtained from guinea pigs four weeks after ultrasound microbubble (USMB) treatment. Representative images in panels **(A–I)** are labeled with phalloidin, panels **(A’–I’)** show the merged DAPI staining images. Immunofluorescence staining shows the nuclei (blue, DAPI) and filamentous actin (green, phalloidin). Scale bars = 50 μm; DAPI, 4,6-diamidino-2-phenylindole; RWS, round window soaking with microbubbles treatment; USM, ultrasound microbubble treatment.

To determine if the USMB treatment cause cellular damage inside the cochlea, a TUNEL-assay was performed in the cochlear structures 24 h after USMB treatment. TUNEL-positive cells were nearly absent in the organ of Corti and spiral ganglion of both the USM and control groups (**Figure 10**). Cochlear sensory epithelial surface preparations obtained from guinea pigs four weeks after USMB treatment showed no significant hair cell damage (**Figure 11**). Taken together, these data suggest that the current protocol for application of USMB for 3 or 5 courses would not damage the receiver’s hearing or their cochlear structure.

## Discussion

The permeability of the RWM can directly reflect the efficacy of inner ear drug delivery *via* RWM transit. In the work reported here, we demonstrated that the sonoporation-enhanced RWM permeability changes may depend on the number of USMB courses, with the highest delivery efficiency observed immediately after USMB treatment. This is followed by a gradual decay in the delivery but a prolonged enhancement still remains for at least 72 h. Concerning the question of whether different temporal profiles of USMB treatments would impact on the measured delivery amounts between USM-3 and USM-5 groups (3 min vs. 5 min), the time difference of 2 min was considered negligible because both groups reached a similar (∼7.5-fold) transmembrane delivery efficiency within 10 min after USMB treatments. We demonstrated that the sonoporation-enhanced permeability change in the RWM may already reach a plateau with a course consisting of between 3 to 5 exposures.

Further exploration of the surface ultrastructure of the RWM after USMB treatment revealed that the MB cavitation and sonoporation effects on the outer epithelial layer also depended on the course of irradiations. The area of epithelial membrane disruption, the membranous pore formation, the gaps or defects between adjacent cells, and the depth of the epithelial breakage were more severe in the group receiving 5 exposures than in the group receiving 3 exposures. These findings were consistent with the results for the USM-3 and USM-5 groups for the biotin-FITC delivery efficiency. As mentioned earlier, although RWM transit may involve several cellular processes, the outer epithelial layer is considered the main barrier to the passage of substances and is directly responsible for membrane permeability (Goycoolea, 2001; Wang et al., 2012; Liu et al., 2013). Our findings in the current study also support this viewpoint, as the USMB-induced permeability enhancement was associated with the physical breakdown of the main barrier, either by disruption of the tight junctions or the creation of membranous holes on the outer epithelial surface of the RWM.

We were the first to extend the application of USMBs to inner ear drug delivery (Shih et al., 2013). In our previous study using confocal laser scanning microscopy, we demonstrated that a fluorescent tracer, when passing through the RWM after USMB treatment, manifested within the cytoplasm of the outer epithelial layer. In addition, this tracer staining was co-localized with actin in the apposed plasma membranes between epithelial cells, i.e., at the cell boundary where tight junctions are formed with the closest contact between adjacent cells (Shih et al., 2013). In this study, the SEM and TEM observations showed that US-induced MB cavitation and sonoporation of the RWM resulted in membrane pore formations and disruption of the continuity and junctions of the outer epithelium, in agreement with our previous histological confocal imaging findings (Shih et al., 2013).

A characteristic of sonoporation treatment is a transient disruption of the cell membrane and an increase in membrane permeability due to acoustic MB cavitation (Mehier-Humbert et al., 2005). This process generates membrane pores ranging in diameter from hundreds of nanometers to microns and is highly associated with the volume expansion, contraction, fragmentation, and collapse of MBs (Kudo et al., 2009; Wang et al., 2013; Fan et al., 2014). Moreover, the change in cell membrane permeability is directly associated with the pore distribution that results from MB cavitation-induced membrane rupture and depends on the ultrasound irradiation applied (Zhou et al., 2012). Our current study revealed an association between sonoporation, membrane permeability, and cavitation-targeted structural changes and is the first to reveal the ultrastructural changes of the RWM after USMB exposure.

The vital roles of the outer epithelium in controlling the permeability of the RWM and subsequent regulation of transport have been described in previous studies (Nomura, 1984; Goycoolea et al., 1988; Suzuki et al., 2003; Wang et al., 2012; Liu et al., 2013). Damaging the outer epithelium with collagenase digestion or phenol treatment were shown to facilitate the delivery of viral vectors (Suzuki et al., 2003; Wang et al., 2012). Interestingly, even after this type of localized enzymatic manipulation had disrupted and eliminated most of the epithelial cells, experimental samples taken 3 or 4 weeks later showed complete recovery and could not be distinguished from the untreated controls (Wang et al., 2012), indicating the high regenerative capacity of epithelial cells. The basement membrane, positioned between the epithelial cells and connective tissue, plays a crucial role in wound healing and in the remodeling process following tissue injury (Brown et al., 2006). The USMB protocol described in the current study showed various degrees of damage that perturbed the outer epithelium and enhanced RWM permeability. However, the treatment still preserved the integrity of the basement membrane, thereby protecting the RWM from sonoporation damage and ensuring its subsequent regeneration.

The underlying mechanisms of USMB treatment have been investigated and involve US-induced MB cavitation that increases the permeability of the targeted cell membranes and capillaries to drugs (Pitt et al., 2004). The acoustic cavitation of MBs can be further classified into stable and inertial cavitation: the former generates a microstreaming and the latter produces a shock wave. An asymmetrical collapse of MBs can even produce a microjet that moves at sonic speed toward the cell surface, accompanied by a shear stress that creates transient but nonlethal micropores in the cell membrane to facilitate the passage of the drug or gene (Pitt et al., 2004; Hernot and Klibanov, 2008). In our related experiments, we have confirmed that US sonication at a suitably diluted MB concentration and a power density of 3 W/cm^2^, as set in this study, induces a cavitation that arises predominantly from the inertial type (unpublished data). Because the current protocol of applying USMBs to an animal’s tympanic cavity required placement of the ultrasound transducer 5 mm away from, but facing, the RWM (Shih et al., 2013), the additional sonoporation effect evoked by direct irradiation may be combined with the effect of MB cavitation, as shown in this study.

The thermal effect occurring during USMB administration is an important issue. In general, a temperature rise of 1.0°C –1.5°C over an indefinite time interval is not considered a safety concern for non-obstetric examinations (Ter Haar, 2011; Harris et al., 2016). A previous report showed the following logarithmic relationship between temperature elevation and the exposure time needed to produce adverse biological effects in animal fetuses for temperatures below 43°C: the necessary exposure time was reduced by a factor of four for every 1°C increase in temperature (Miller and Ziskin, 1989). Applying this logarithmic rule, the maximum safe exposure time would be 4 min for a temperature elevation of 4°C, 16 min for 3°C, 64 min for 2°C, and 256 min for 1°C (Harris et al., 2016). A reduction from a 256 min maximum exposure time to 120 min has been suggested as a safety precaution to reflect the present limited knowledge about possible subtle thermal bioeffects. However, the results from the present study showed that the temperature on the cochlear basal turn increased by 2.0°C–2.7°C and that of the cochlear apex increased by 1.2°C–1.8°C during sonication with 3–5 courses of USMBs, indicating that temperatures are unlikely to extend beyond the normal physiological range. Our investigation of hearing assessment and our histological examinations, including ABRs, DPOAE, surface preparations, and TUNEL assays, also indicated that this USMB technique is not harmful when applied as a method for inner ear drug delivery. Nevertheless, taking ultrasonic thermal safety precautions against any adverse bioeffects is always imperative, especially when a sensory organ is exposed to a prolonged duration of elevated temperature.

The conveyance of substances through the RWM to the inner ear primarily relied on a passive process, while active transport was assumed to be in charge of larger molecules and particles (Goycoolea, 2001; Salt and Plontke, 2005). When placed on the RWM, the substances may undergo nonspecific pinocytosis or pass through different channels between epithelial cells to traverse through the cytoplasm, undergo phagocytosis by the connective tissue cells, and then either penetrate blood vessels in the connective tissue layer or flow further into the perilymph (Goycoolea and Lundman, 1997; Duan and Chen, 2009). In addition to the physical alterations observed in the ultrastructural evaluations by TEM and SEM, the interactions between cavitation events and targeted cells also evoke a series of spatiotemporal molecular responses and biological effects that provide a temporary and reversible time window for drug delivery and repair of cavitation-induced membrane perforations (Qin et al., 2018). For example, receptor and caveolin-mediated endocytosis, a specific and active route for drug delivery, can be stimulated by acoustic cavitation (Zeghimi et al., 2015). The Ca^2+^-gated ion channels are transiently activated and the intracellular Ca^2+^ transients can be detected after cavitation; these responses are temporally correlated with the occurrence of sonoporation (Fan et al., 2014; Qin et al., 2018). Changes in membrane potential, cytoskeleton dynamics, and in the production of reactive oxygen species were also reported after sonoporation (Qin et al., 2018).

The mechanisms for resealing USMB-mediated membrane perforations may rely on exocytosis and endocytosis (Fan et al., 2014; Qin et al., 2018). A large membrane perforation can trigger exocytosis of intracellular vesicles that, in turn, reseal the perforation with fused exocytotic vesicles. A small membrane perforation can be eliminated *via* endocytosis initiated by Ca^2+^ influx. This study focused on investigating sonoporation-induced ultrastructural alterations that may explain in part the mechanism of the observed permeability changes. Further exploration of the molecular biological mechanisms involved in cavitation-regulated membrane repair and prolonged permeability will be needed in the future.

## Conclusion

Our findings demonstrated that the application of USMBs for the delivery of drugs to the inner ear was a safe, feasible, and effective approach to enhance RWM permeability. Scanning and transmission electron microscopy revealed the morphological changes that corresponded to the increased RWM permeability and indicated that the enhanced RWM permeability can result directly from MB cavitation-induced disruption of the barrier formed by the outer epithelial layer of the RWM. The sonoporation effects on the targeted cells and the prolonged effects on membrane permeability were dependent on the USMB irradiation course. Although epithelial cells were transiently disrupted by cavitation, their basement membranes remained intact and could completely recover within one month. Our findings provide a better understanding of how to develop therapeutic USMBs for inner ear drug and gene delivery for use in future clinical trials.

## Data Availability Statement

All datasets generated for this study are included in the article.

## Ethics Statement

This study was carried out in accordance with relevant institutional and national guidelines for the care and use of laboratory animals. The experimental protocol was approved by the Institutional Animal Care and Use Committee of the National Defense Medical Center, Taipei, Taiwan (ethic code: IACUC-15-165; permission date: 1 August 2015).

## Author Contributions

Y-CL completed the main experiment and wrote the first draft of the paper. H-CC and H-KC participated in planning and analyzed the data. Y-YL, C-YK, and HW performed the surgery. C-LH assisted with statistical analysis. C-PS and C-HW initiated the project and designed the experiments. C-HW edited and revised the manuscript. All authors have reviewed the final version of the manuscript and approved it for publication.

## Funding

This work was supported in part by grants from the Ministry of Science and Technology, Taiwan (MOST107-2314-B-016-027 and MOST 108-2314-B-016-038 to C-PS and MOST107-2314-B-663-001-MY3 to C-HW), the Tri-Service General Hospital grants (TSGH-C108-011 to C-PS and TSGH-C107-009 to C-HW), the Taichung Armed Forces General Hospital grant (108A14 to C-HW), and the Teh-Tzer Study Group for Human Medical Research Foundation (A1041001 and A1061019 to C-HW).

## Conflict of Interest

The authors declare that the research was conducted in the absence of any commercial or financial relationships that could be construed as a potential conflict of interest.
